# Coexistence of lobular granulomatous mastitis and breast cancer: Case report and case-based literature review

**DOI:** 10.1016/j.ijscr.2025.111628

**Published:** 2025-07-09

**Authors:** Mina Akbari Rad, Fereshte Sheybani, Masoumeh Gharib, Elahe Aghel, Maryam Emadzadeh, Mahdieh Mottaghi

**Affiliations:** a Clinical Research Development Unit, Ghaem Hospital, Mashhad University of Medical Sciences, Mashhad, Iran; b Department of Infectious Diseases and Tropical Medicine, Faculty of Medicine, Mashhad University of Medical Sciences, Mashhad, Iran; c Department of Pathology, Faculty of Medicine, Mashhad University of Medical Sciences, Mashhad, Iran; d Cancer Research Center, Omid Hospital, Mashhad University of Medical Sciences, Mashhad, Iran

**Keywords:** Granulomatous mastitis, Breast neoplasms, Ductal carcinoma in situ, Invasive ductal carcinoma, Case report

## Abstract

**Introduction and importance:**

This study aims to provide insights into the characteristics of patients who experience both conditions of lobular granulomatous mastitis (LGM) and breast carcinoma. This patient was the only case among our 246 consecutive patients with LGM who experienced both LGM and malignancy.

**Case presentation:**

A 46-year-old Persian woman was diagnosed with left-sided LGM via core-needle biopsy, which resolved following two years of prednisone and subsequent methotrexate therapy. Three months after remission, she developed stiffness in the contralateral breast, and biopsy revealed grade 3 invasive ductal carcinoma that was estrogen receptor (ER)-positive, progesterone receptor (PR)-negative, and HER2 (1+).

**Clinical discussion:**

The prevalence of coexisting LGM and breast cancer among LGM cases was 0.41 %. In our review of 20 patients, LGM occurred prior to breast cancer in nine cases (45 %), concurrently in nine cases (45 %), and subsequent to breast cancer in two cases (10 %). Among the 20 reviewed cases, invasive ductal carcinoma (IDC) was the most frequently identified malignancy, observed in 15 patients (75 %), while ductal carcinoma in situ (DCIS) was reported in five cases (25 %). Hormone receptor positivity (estrogen and/or progesterone receptor) was noted in 11 patients (55 %), and HER2 overexpression was present in seven cases (35 %).

**Conclusion:**

New breast findings in individuals previously diagnosed with LGM should not be readily interpreted as a disease recurrence. To minimize the risk of misdiagnosis, bilateral assessment—including bilateral mammography or biopsy—is recommended, particularly in older patients, postmenopausal women, those with recurrent episodes, or when the contralateral breast is involved.

## Highlights

• The incidence of coexisting LGM and breast cancer was 1.18 per 1000 person-years.
• The prevalence of this coexistence was 0.57% (1 in 176 patients).
• New breast findings in LGM patients do not always indicate recurrence.
• Full evaluation via mammography or biopsy is key to avoid misdiagnosis in high-risk groups.
• Pay close attention to older, postmenopausal, or recurrent cases.

## 1 Introduction

Lobular granulomatous mastitis (LGM) is a chronic inflammatory disease of the breast that is often misdiagnosed as breast cancer [1]. While some patients initially present with a breast mass and mild inflammatory changes, others mimic inflammatory breast cancer [2,3]. The optimal therapeutic approach for LGM should be individualized based on clinical manifestations and disease severity [4]. Previous studies suggested that combination therapy with methotrexate (MTX) and corticosteroids offers superior efficacy [4,5].

Recurrence rates of LGM are high, affecting 18 % to 50 % of cases [2,[6], [7], [8]], with 3–8 % involving the contralateral breast [6,[8], [9], [10]]. Despite being considered a benign condition, co-occurrence of LGM and breast cancer has been rarely described [[11], [12], [13], [14], [15], [16], [17], [18], [19], [20], [21]].

At our internal medicine clinic in a tertiary teaching hospital, all pathologically confirmed cases of LGM have been documented from January 2015 to December 2021. Among 246 consecutive patients, only one case of coexisting LGM and breast cancer was identified. This corresponds to an incidence rate of 1.18 per 1000 person-years across a cumulative follow-up of 849 person-years and a prevalence of 0.57 % (1 in 176 patients). These findings underscore the rarity of the occurrence of LGM and breast malignancy in a single patient.

Herein, we present a patient with LGM who subsequently experienced recurrence of symptoms in contralateral breast, which was diagnosed with invasive ductal carcinoma. This case report has been reported in line with the SCARE 2025 checklist [22]. Additionally, we conducted a review of existing literature to explore the potential association between LGM and neoplastic breast lesions. This study aimed to address two primary questions: 1) Can LGM coexist with malignancy? 2) What are the characteristics of patients with the coexistence of breast cancer and LGM?

## 2 Presentation of case

A 46-year-old premenopausal Iranian woman presented to the internal medicine outpatient clinic with complaints of a breast mass, erythema, and swelling. On physical examination, a tender mass was palpated in the lateral quadrant of the left breast near the areola, without evidence of axillary lymphadenopathy. She had a history of hypothyroidism, for which she was taking levothyroxine at a dose of 50 micrograms daily.

**Fig. 1:**
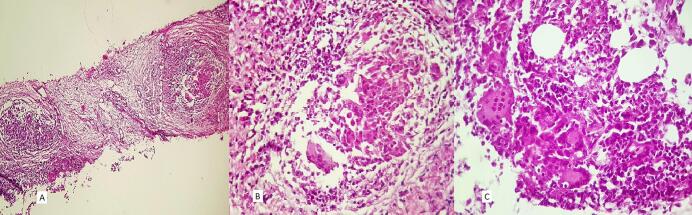
Core-needle biopsy of the breast with a granulomatous formation, composed of epithelioid histiocytes and giant cells, encircling empty spaces (H&E, A (100×), B (400×), C (400×)).

**Fig. 2:**
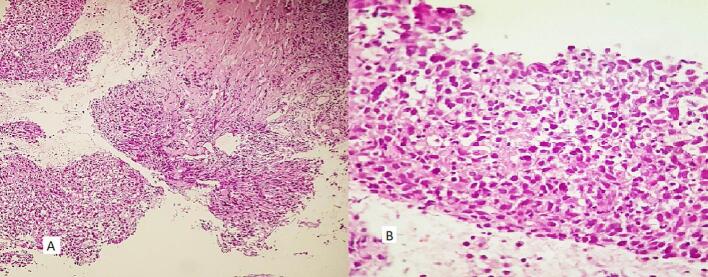
Core-needle biopsy of the breast with sheets of atypical epithelial cells with scant ductal formations (A, B) (H&E, A (40×), B (400×)).

**Fig. 3:**
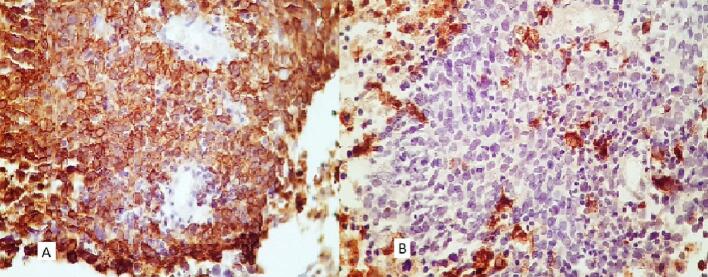
Positive CK staining in carcinoma cells (A); negative CD68 staining in carcinoma cells (B) (400×).

The patient was diagnosed with idiopathic LGM (Fig. 1). Initial treatment with prednisone at a dose of 15 mg daily, later increased to 20 mg daily, resulted in no significant clinical improvement. However, prednisone led to weight gain, peripheral edema, and elevated blood glucose levels within the prediabetic range. Consequently, following nine months of treatment, prednisolone was tapered, and MTX was initiated at a dose of 12.5 mg per week. MTX led to complete resolution of the breast mass over a two-year period. However, three months after complete remission of LGM, the patient presented with stiffness in the contralateral breast. Physical examination revealed a non-tender mass in the upper lateral quadrant of the right breast. A core-needle biopsy of the breast mass showed involvement of the breast parenchyma by atypical cells with moderate pleomorphism, mitosis, and focal necrosis in the background of mastitis, suggestive of invasive carcinoma grade 3 (Fig. 2). Immunohistochemical staining demonstrated positive expression of cytokeratin (CK) and a negative result for cluster of differentiation 68 (CD68) (Fig. 3). The patient was referred to an oncologist for further evaluation. Radiological staging revealed an ill-defined mass measuring approximately 32 × 26 mm in the central region of the right breast, along with a few variably sized lymph nodes in the right axilla, with the largest measuring approximately 14 × 10 mm. No evidence of osseous, hepatic, or pulmonary metastases was observed. Immunohistochemical analysis of the tumor demonstrated estrogen receptor (ER) positivity in 50 % of tumor cells, while progesterone receptor (PR) and human epidermal growth factor receptor 2 (HER2) were negative (scored 1+). The patient subsequently underwent neoadjuvant chemotherapy, receiving four cycles of dose-dense doxorubicin (60 mg/m^2^) and cyclophosphamide (600 mg/m^2^), followed by four cycles of dose-dense paclitaxel (175 mg/m^2^). As the tumor responded with a reduction in size, she was scheduled to receive surgery, radiotherapy, and hormone therapy. At the time of manuscript submission, the patient was alive, with the most recent follow-up conducted in December 2024.

## 3 Case-based review

In this review of 20 patients with lobular granulomatous mastitis (LGM) and breast carcinoma, cases were categorized as follows: LGM preceding cancer (n = 9, 45 %) (Table 1), concurrent with cancer (n = 9, 45 %) (Table 2), and following cancer (n = 2, 10 %) (Table 3).

**Table 1:** Part A. Summary of patients with lobular granulomatous mastitis prior to breast cancer: Data on LGM. Part B, Summary of patients with lobular granulomatous mastitis prior to breast cancer: Data on malignancy.

Part A												
Case	First author, year	Country	Age, years	Menstruation status	Parity	Initial presentation	Diameter*, mm	LGM and malignancy lateralization	Recurrent pathology proved LGM	OCP use	Sampling method	Treatment for LGM
Case 1 [15]	Mazlan, 2012	Malaysia	34	Premenopausal	-	Abscess, swelling	-	Ipsilateral LGM: R Malignancy: R	Yes	No	-	Pred, I&D
Case 2 [21]	Zangouri, 2022	Iran	38	Premenopausal	-	M	88	Ipsilateral LGM: L Malignancy: L	Yes	-	Tru-cut biopsy for LGM	Pred, MTX
Case 3 [11]	Yoshida, 2023	Japan	34	-	-	M, E	40	Ipsilateral LGM: L Malignancy: L	No complete improvement on treatment	-	FNA and CNB for LGM; excisional biopsy for malignancy	Pred, excision
Case 4 [14]	Abdulwahid, 2024	Iraq	30	Premenopausal	3	M, abscess, P, swelling, NR, LAP	-	Ipsilateral LGM: L Malignancy: L	Yes	No	Excisional biopsy	AB, Pred, MTX, I&D, excision
Case 5 [52]	Akbari, 2024	Iran	37	-	-	M, bloody ND after 18 months	30	Ipsilateral LGM: L Malignancy: L	Yes	-	CNB for LGM, repeated CNB for malignancy	Observation
Case 6 [52]	Akbari, 2024	Iran	40	-	-	M, abscess, P, E, draining fistula, bloody ND after 6 months	50	Ipsilateral LGM: R Malignancy: R	No obvious improvement on treatment	-	CNB for LGM, repeated CNB for malignancy	Non-surgical treatment
Case 7 [39]	Mohammadi-Zavieh, 2024	Iran	46	Premenopausal	2	M	-	Ipsilateral LGM: R Malignancy: R	No	No	CNB	Pred
Case 8 [39]	Mohammadi-Zavieh, 2024	Iran	41	Premenopausal	-	M	22	Contralateral LGM: L Malignancy: R	No	No	CNB	Low-dose corticosteroids
Case 9	Our case	Iran	46	Premenopausal	3	M, E, Swelling	25	Contralateral LGM: Left Malignancy: R	No	No	CNB	Pred, MTX
*We considered the largest diameter of mass/collection based on sonographic findings or physical examination at presentation. Characteristics with unknown status are indicated by a dash (-). Abbreviations in alphabetical order, AB: antibiotics, CNB: core-needle biopsy, E: erythema, FNA: fine needle aspiration, I&D: incision and drainage, L: left, LAP: lymphadenopathy, LGM: lobular granulomatous mastitis, M: mass, MTX: methotrexate, ND: nipple discharge, NR: nipple retraction, OCP: oral contraceptive pill, P: pain, Pred: prednisone, R: right, y: years.												


Part B									
Case	FH of breast cancer	Duration between diagnosis of LGM and malignancy	Evidence of calcification on breast imaging	Type of breast cancer	Grade of cancer	Staining/IHC	Axillary LN metastasis	Distal metastasis	Outcome
Case 1	No	Eight years	-	Invasive ductal carcinoma	4	ER (+), PR (+), HER2 (−)	-	Vertebrae, brain	Patient declined treatment and expired after 6 months.
Case 2	-	One year	-	Invasive ductal carcinoma	3	-	-	-	-
Case 3	No	Nine weeks	-	DCIS	Low grade	ER (+), PR (+), E-cadherin, Ki-67 labeling index: 10.5 %	-	-	Total mastectomy, hormone therapy
Case 4	Paternal aunt	Two years	Micro calcifications on mammography	DCIS	High grade	ER (+)	Negative	No	Mastectomy, SLNB, hormone therapy
Case 5	-	Eighteen months	-	DCIS	High grade	-	-	-	Management of DCIS
Case 6	No	Three months	Micro calcifications on mammography	Invasive ductal carcinoma	-	-	-	-	Surgery
Case 7	No	Four years	No calcification	Invasive ductal carcinoma	-	ER (−), PR (−), HER2 (−)	-	No	Neoadjuvant chemotherapy, mastectomy, SLNB, post-mastectomy radiotherapy
Case 8	No	Thirteen years	No calcification	Invasive ductal carcinoma	-	ER (+), PR (+), HER2 (3+)	-	No	Adjuvant chemotherapy, breast conserving, SLNB, radiotherapy
Case 9	Paternal aunt	Three months	No calcification	Invasive ductal carcinoma	Grade 3	ER (+), PR (−), HER2 (1+), CK (+), CD68 (−)	Yes (right axilla)	No	Neoadjuvant chemotherapy, mastectomy, radiotherapy, and hormone therapy
Characteristics with unknown status are indicated by a dash (-). Abbreviations in alphabetical order, DCIS: ductal carcinoma in situ, ER: estrogen receptor, FH: family history, HER2: human epidermal growth factor receptor 2, IHC: immunohistochemistry, Ki67: proliferation marker used in cancer grading, LN: lymph node, PR: Progesterone receptor, SLNB: sentinel lymph node biopsy.

**Table 2:** Part A. Summary of patients with lobular granulomatous mastitis concurrent with breast cancer: Data on LGM. Part B. Summary of patients with lobular granulomatous mastitis concurrent with breast cancer: Data on malignancy.

Part A												
Case	First author, year	Country	Age, years	Menstruation status	Parity	Initial presentation	Diameter*, mm	LGM and malignancy lateralization	Recurrent pathology proved LGM	OCP use	Sampling method	Treatment for LGM
Case 10 [12]	Limaie, 2013	Tunisia	77	Postmenopausal	-	M, P, NR	24	Ipsilateral LGM: R Malignancy: R	No	-	CNB	–
Case 11 [19]	Kaviani, 2017	Iran	48	-	-	Right: M, edema, E, NR, peau d'orange, LAP Left: M	Left: 35, right: 50	Contralateral LGM: R Malignancy: L	No	No	CNB	Observation
Case 12 [16]	Çalış, 2018	Turkey	77	Postmenopausal	-	P, edema, peau d'orange, LAP	0	Ipsilateral LGM: R Malignancy: R	No	-	CNB for LGM; excisional biopsy for malignancy	–
Case 13 [17]	Özşen, 2018	Turkey	35	-	-	M, abscess, swelling	34	Ipsilateral LGM: R Malignancy: R	No obvious improvement on treatment	-	Tru-cut biopsy for LGM; excisional biopsy for malignancy	AB Pred, excision
Case 14 [20]	Oddó, 2019	Chile	44	-	-	M, abscess, P, inflammation	70	Ipsilateral LGM: L Malignancy: L	No	-	CNB	AB, Pred, MTX, I&D, excision
Case 15 [13]	Evans, 2021	Australia	39	-	2	Left: M, P Right: Normal	60	Contralateral LGM: L Malignancy: R	No, history of breast abscess	-	CNB	AB, I&D, excision
Case 16 [18]	Zhu, 2023	China	51	Postmenopausal	-	M, edema	-	Ipsilateral LGM: L Malignancy: L	No	-	CNB	–
Case 17 [18]	Zhu, 2023	China	50	Perimenopause	-	M, P, E, swelling, peau d'orange, NR, comedogenic ND	-	Ipsilateral LGM: L Malignancy: L	No	-	Excisional biopsy	Anti-inflammatory, I&D, excision
Case 18 [18]	Zhu, 2023	China	45	Perimenopause	-	M, P, E, NR, swelling, NR, comedogenic ND	-	Ipsilateral LGM: L Malignancy: L	No	-	CNB	I&D, excision
*We considered the largest diameter of mass/collection based on sonographic findings or physical examination at presentation. Characteristics with unknown status are indicated by a dash (-). Abbreviations in alphabetical order, AB: antibiotics, CNB: core-needle biopsy, E: erythema, I&D: incision and drainage, LAP: lymphadenopathy, LGM: lobular granulomatous mastitis, M: mass, MTX: methotrexate, ND: nipple discharge, NR: nipple retraction, OCP: oral contraceptive pill, P: pain, Pred: prednisone.												


Part B								
Case	FH of breast cancer	Evidence of calcification on breast imaging	Type of breast cancer	Grade of cancer	Staining/IHC	Axillary LN metastasis	Distal metastasis	Outcome
Case 10	No	No calcification	Invasive ductal carcinoma	2	ER (+), PR (+), HER2 (0)	Positive	-	Radical mastectomy with homolateral axillary lymphadenectomy
Case 11	No	Calcification on sonography	Invasive ductal carcinoma	-	-	No	No	Palliative chemotherapy, breast-conserving surgery, radiotherapy, Hormone therapy
Case 12	-	Calcification on mammography	Simultaneous invasive ductal carcinoma and DCIS	High grade	ER (−), PR (−), HER2 (+++)	Positive	-	Radical mastectomy, adjuvant chemotherapy
Case 13	-	-	DCIS	-	CD10 (+), p63 (+), CK5/6 (+)	-	-	Patient was lost to next follow ups.
Case 14	No	No calcification	DCIS	2	ER (intensely positive), PR (intensely positive)	-	-	Total mastectomy
Case 15	No	Microcalcifications on mammography	CNB: high grade DCIS Excisional biopsy: invasive ductal carcinoma	3	PR (++), HER2 (−)	Positive	No	Wide local excision, SLNB and axillary dissection, adjuvant chemotherapy, radiotherapy, hormone therapy
Case 16	-	-	DCIS	High grade	ER (−), PR (−), HER2 (1+), 6 around ductal myoepithelium (+), p63 (+), CK5/6 (+)	-	-	Breast conserving surgery, SLNB
Case 17	-	Dense calcification on mammography	DCIS	High grade	ER (−), PR (−), HER2 (3+), 6 around ductal myoepithelium (+), p63 (+), CK5/6 (+)	-	-	Breast-conserving surgery, SLNB
Case 18	-	-	DCIS	Low grade	ER (+), PR (+), HER2 (3+), 6 around ductal myoepithelium (+), p63 (+), CK5/6 (+)	-	-	Breast-conserving surgery, SLNB
Characteristics with unknown status are indicated by a dash (-). Abbreviations in alphabetical order, CD10: cluster of differentiation 10, CK5/6: cytokeratin 5/6, CNB: core-needle biopsy, DCIS: ductal carcinoma in situ, ER: estrogen receptor, FH: family history, HER2: human epidermal growth factor receptor 2, IHC: immunohistochemistry, LN: lymph node, PR: progesterone receptor, SLNB: sentinel lymph node biopsy, p63: tumor suppressor protein.

**Table 3:** Part A. Summary of patients with lobular granulomatous mastitis subsequent to breast cancer: Data on LGM. Part B. Summary of patients with lobular granulomatous mastitis subsequent to breast cancer: Data on malignancy.

Part A											
Case	First author, year	Country	Age, years	Menstruation status	Parity	Initial presentation	Diameter, mm	LGM and malignancy lateralization	OCP use	Sampling method	Treatment for LGM
19 [39]	Mohammadi-Zavieh, 2024	Iran	42	Premenopausal	2	Pain, mass, periareolar erythema, nipple inversion	50 × 29 × 22 mm	Cancer: right, LGM: left	Short-term use ~20 years ago	Core needle biopsy	NSAID (Naproxen), surgery (open drainage), biopsy, continued medical therapy
20	Mahmood, 2025	Pakistan	45	Premenopausal	7	Mass	25.7 × 12.5 × 25.0 mm	LGM bilateral, malignancy left	-	Ultrasound-guided core biopsy	Pred, methotrexate
Characteristics with unknown status are indicated by a dash (-). Abbreviations in alphabetical order, LGM: lobular granulomatous mastitis, mm: millimeters, NSAID: non-steroidal anti-inflammatory drug, OCP: oral contraceptive pill, Pred: prednisone.											


Part B								
Case	FH of breast cancer	Duration between malignancy and diagnosis of LGM	Evidence of calcification on breast imaging	Type of breast cancer	Grade of cancer	Staining/IHC	Axillary LN metastasis	Distal metastasis
1	No	LGM occurred 2 years after breast cancer	No	Invasive ductal carcinoma	-	ER (+), PR (+), HER2 (3+)	-	No
2	No	LGM occurred after cancer diagnosis, during 2nd cycle of chemotherapy	No	Invasive ductal carcinoma	Grade 3	ER (+), PR (−), HER2 (3+)	Left axillary lymphadenopathy	No
Characteristics with unknown status are indicated by a dash (-). Abbreviations in alphabetical order, ER: estrogen receptor, FH: family history, HER2: human epidermal growth factor receptor 2, IHC: immunohistochemistry, LN: lymph node, LGM: lobular granulomatous mastitis, PR: progesterone receptor.								

### 3.1 LGM prior to breast cancer

Patients were predominantly premenopausal (7/9, 78 %) with a median age of 37 years (range 30–46). Parity ranged from 0 to 3 (median: 2). All nine (100 %) presented with a palpable breast mass; other symptoms included swelling (7/9, 78 %), pain (5/9, 56 %), erythema (3/9, 33 %), and abscess formation (3/9, 33 %). Lesion diameters ranged 25–88 mm (median: 40 mm). Lateralization was ipsilateral in seven (77.8 %) and contralateral in two (22.2 %). Four women (4/9, 44 %) experienced recurrent LGM. No patient used hormonal contraception.

All patients underwent tissue confirmation by core-needle biopsy (7/9, 78 %) or excisional biopsy (2/9, 22 %). Initial therapy included systemic corticosteroids alone in four (44 %) and combined with methotrexate in three (33 %), incision and drainage in two (22 %), and antibiotics in one (11 %). Complete LGM resolution required a median of 18 months (range 9–24).

Interval to malignancy ranged from 9 weeks to 13 years (median: 18 months). Histology revealed invasive ductal carcinoma (IDC) in six (67 %) and DCIS in three (33 %). Tumors were high-grade (grade 3–4) in four (44 %) and low-grade in two (22 %); receptor positivity (ER and/or PR) was noted in seven (78 %), with HER2 overexpression in three (33 %). Distant metastases occurred in two (22 %); one patient (11 %) declined treatment and died within six months.

### 3.2 Concurrent LGM and breast cancer

Median age was 48 years (range 35–77); five (56 %) were postmenopausal. Presentations included peau d'orange (3/9, 33 %), lymphadenopathy (4/9, 44 %), erythema (4/9, 44 %), and abscess/draining fistula (2/9, 22 %). Diameters spanned 0–70 mm (median 35 mm). Ipsilateral involvement occurred in seven (78 %); contralateral in two (22 %). All diagnoses used core-needle or Tru-cut biopsy.

IDC was identified in seven (78 %) and DCIS in two (22 %). Receptor profiles were heterogeneous: ER/PR-negative in three (33 %), HER2-positive in four (44 %), and ER/PR positive in two (22 %). Axillary metastases were present in three (33 %); no distant spread was seen (0 %).

### 3.3 LGM following breast cancer

Both women were premenopausal (2/2, 100 %), aged 42 and 45. LGM onset occurred two years' post-cancer in one case and during chemotherapy in the other. Presenting features included periareolar erythema and nipple inversion (2/2, 100 %) and palpable mass (2/2, 100 %). Imaging-guided core-needle biopsy confirmed LGM in all (100 %). Management combined NSAIDs (2/2, 100 %), corticosteroids (1/2, 50 %), methotrexate (1/2, 50 %), and surgical drainage (1/2, 50 %). Both had prior IDC; one (50 %) was ER+/PR+/HER2+, the other ER+/PR–/HER2+. Axillary nodal involvement was seen in one (50 %); neither experienced distant metastases (0 %).

## 4 Discussion

We presented a case of a 46-year-old woman diagnosed with LGM in her left breast, which was successfully treated with corticosteroids and MTX. Subsequently, she developed an invasive ductal carcinoma in her right breast, which was ER positive, PR negative, and HER2 negative. Staging radiological evaluations revealed no evidence of distant metastases. While LGM is generally considered a benign condition, multiple reports have documented its occurrence alongside breast carcinoma. Overall, our review provides valuable insights into the rare but clinically significant association between LGM and breast cancer.

One notable observation is the median age of 40 years among the reported cases, with a considerable proportion occurring in postmenopausal individuals. The median age of 40 years suggests that patients affected by both LGM and breast cancer tend to be older, which may be unexpected given that LGM is typically considered a condition affecting younger women, often during their childbearing years.

Calcifications on breast imaging are also a common finding, observed in the majority of cases where imaging data were available. This highlights the potential utility of radiological assessment in identifying suspicious lesions and guiding further diagnostic evaluation in patients with LGM.

While some cases demonstrated a subsequent diagnosis of breast carcinoma either in the same breast or in the contralateral breast of patients with LGM [11,14,15,21], other cases described an incidental finding of ductal breast carcinoma in patients initially diagnosed with LGM in the same breast [12,13,[16], [17], [18], [19], [20]]. Breast cancer involvement predominantly occurred in the same breast as LGM. However, the occurrence of contralateral breast involvement in a small proportion of cases underscores the need for bilateral assessment and management strategies in these patients.

The histological subtypes of breast cancer observed in our review varied, with a notable proportion exhibiting features of ductal carcinoma in situ (DCIS). However, immunohistochemistry staining revealed heterogeneous expression patterns of hormone receptors and HER2, further underscoring the molecular heterogeneity of LGM-associated breast cancer.

It remains unclear whether the co-occurrence of LGM and breast cancer is incidental or reflects a causal link. The rarity of reported cases, along with the inconsistency in lesion lateralization—whether the malignancy arises in the same or contralateral breast affected by LGM—diminishes the likelihood of a direct causal association. However, the rare co-occurrence of these conditions does not necessarily rule out causality, as it may be due to overall low prevalence of LGM. Two hypotheses could potentially explain the causal link between LGM and malignancy: (1) the granulomatous response occurring as a secondary reaction to breast carcinoma, which may have been misdiagnosed as LGM and (2) the chronic inflammatory condition induced by LGM, possibly predisposing to dysplasia and subsequent development of breast cancer. These possibilities highlight the need to explore potential mechanisms links between sustained inflammatory responses and breast cancer.

To support the first hypothesis, malignancy-related granulomatous inflammation—also referred to as a “sarcoid-like reaction”—was first described in 1911 [23,24]. This reaction has been observed in approximately 4.4 % of various carcinomas, including dysgerminoma/seminoma, Hodgkin lymphoma [6], colorectal carcinoma [25], renal cell carcinoma [26], lung cancer [27], hepatocellular carcinoma [28], and breast cancer [27]. The underlying mechanism is thought to involve immunological hypersensitivity to antigens derived from tumor cells, triggering granuloma formation [29]. Antigen-loaded dendritic cells stimulate Interleukin (IL)-12 production and present antigens to CD-4 cells, which then differentiate into T-helper (Th)-1 cells. These activated Th-1CD4+ cells interact with macrophages, inducing interferon-gamma production and subsequent granuloma formation [30].

Additionally, breast cancer is characterized by a significant chronic inflammatory component, marked by the infiltration of lymphocytes and macrophages. Studies have shown that approximately 91 % of patients with invasive ductal carcinoma exhibit high-grade inflammatory immune cell infiltrates, including macrophages, T- and B-lymphocytes [31]. Granuloma formation -in invasive ductal carcinoma was first reported in 1987 and subsequently observed in other breast cancer types as well [[32], [33], [34], [35], [36]]. A review of the literature up to 2013 identified 38 patients diagnosed with breast cancer and sarcoid-like reaction, with ductal breast carcinoma being the most commonly associated subtype [37]. Additionally, recent studies reported development of LGM following treatment for invasive ductal carcinoma [38,39].

Evidence supporting the second proposed mechanism suggests that chronic inflammation induced dysplasia might eventually progress to malignancy. Previous studies have highlighted the role of inflammation in the initiation, progression, and invasion of breast cancer [40,41]. A retrospective case-control study demonstrated a significant association between mastitis and the risk of breast cancer, with a relative risk (RR) of 2.06 among patients with mastitis [42]. Similarly, another retrospective cohort study revealed that patients with mastitis had a substantially higher risk of breast cancer compared to the control group, with an adjusted hazard ratio of 3.71 (95 % confidence interval [CI]: 1.9–7.02) [43]. Consistent with these findings, a population-based cohort study showed that women with non-lactational mastitis had an elevated risk of breast cancer compared to the comparison group, with an adjusted hazard ratio of 1.94 (95 % CI: 1.30–2.90) [44]. Moreover, inflammatory lesions of the breast exhibit many similarities with ER-negative malignant breast tumors, such as low expression levels of ER and activation of similar immune signaling pathways [45].

Furthermore, prior studies have demonstrated significantly elevated levels of plasma IL-17 (p = 0.018) and IL-33 (p < 0.001) in patients with LGM compared to the control group [46,47]. Both Th-17 and IL-33 have been implicated in carcinogenesis [48,49], and elevated plasma levels of these interleukins have been observed in breast cancer as well [[47], [48], [49]].

Given the administration of MTX in the treatment of LGM, it is important to clarify whether there is an association between MTX therapy and risk of breast cancer. Based on current evidence, MTX, despite its immunosuppressive effects, is not associated with increased overall breast cancer risk. A ten-year cohort of 1566 patients with RA who received MTX demonstrated no statistically significant increase in the incidence of breast cancer (2.44 per 1000 person-year; 95 % CI, 0–4.89) [50]. In a cohort study on women with RA and a history of breast cancer, MTX use was not associated with an increased risk of breast cancer recurrence (HR = 1.07; 95 % CI, 0.67–1.69) [51].

However, the limited number of reported cases documenting the coexistence of malignancy and LGM may affect the generalizability of our findings. Moreover, the results from our literature review lack sufficient data to provide a comprehensive demographic profile of LGM patients who are at risk of developing or coexisting with breast carcinoma, due to inconsistencies in the clinical and pathological characteristics reported across the included studies. Thus, we recommend that future research provide comprehensive documentation of breast cancer risk factors, such as a family history of breast cancer in first- or second-degree relatives, nulliparity, and hormone use. Furthermore, collecting detailed data on imaging findings—particularly the presence of calcifications—tumor grade, immunohistochemical staining profiles, axillary lymph node involvement, and evidence of distant metastases would significantly enhance our understanding of the potential relationship between LGM and breast malignancy. Such standardization in reporting could contribute to more robust conclusions and facilitate comparisons across studies.

## 5 Conclusion

Our findings underscore the potential occurrence of breast carcinoma concurrently with or subsequent to the diagnosis of LGM, either in the same or contralateral breast. Regardless of whether this co-occurrence is incidental or reflects a causal relationship, clinicians should remain vigilant to avoid misinterpreting new breast findings in patients with a history of LGM as a recurrence, thereby potentially overlooking an underlying malignancy. Accordingly, it may be prudent to perform bilateral mammography followed by biopsy of any newly detected breast abnormality in patients with LGM, particularly in those of advanced age, postmenopausal status, those with recurrent mastitis, or when symptoms arise in the contralateral breast.

## Consent for publication

The patient is conscious and has consented to the publication of her case.

## CRediT authorship contribution statement

MM: Investigation, writing original draft of the manuscript. FS: Design of the work, analysis and interpretation of data, revising the manuscript. MG: Revising the manuscript regarding breast histopathology. EA: Revising the manuscript regarding breast cancer management. ME: Revising the manuscript regarding methodology, MAR: Conceptualization and project administration. All authors read and approved the final manuscript.

## Consent

The study approval (Code: 4010734) and the ethical consideration for following up of this patient was received. The patient who included in the study signed the informed consent.

## Ethical approval

The study approval (Code: 4010734) and the ethical consideration for following up of this patient was received. The study was ethically approved by the Ethics Committee of the Mashhad University of Medical Sciences (Code: IR.MUMS.MEDICAL.REC.1401.609). The patient who included in the study signed the informed consent.

## Guarantor

MAR and MM had access to the data, and controlled the decision to publish.

## Declaration of Generative AI and AI-assisted technologies in the writing process

Generative AI (ChatGPT, OpenAI, GPT-4, March 2024 version) was used solely to assist with language refinement, grammar correction, and structural clarity during the writing and revision stages of this manuscript. No clinical data analysis, image interpretation, or figure generation was performed by AI. The tool was accessed via a cloud-based platform (chat.openai.com) without plug-ins or fine-tuning. Only de-identified, non-sensitive text from the manuscript was input, fully compliant with GDPR and HIPAA standards; no patient data or images were shared. All AI-assisted content was reviewed, fact-checked, and edited by the authors, who take full responsibility for the final manuscript. Potential algorithmic bias was addressed through manual cross-referencing with established literature. The authors have no financial ties to AI vendors and affirm adherence to ethical research and reporting standards. Sample prompts and outputs are available upon request.

## Funding

There was no funding.

## Declaration of competing interest

There are no competing interests.

## Data Availability

The data used to support the findings of this study are available from the corresponding author upon request.
